# Supplementary material for: ATP-independent substrate recruitment to proteasomal degradation in mycobacteria
Source: Life Sci Alliance. 2023 Aug 10;6(10):e202301923. doi: 10.26508/lsa.202301923 (PMC10415612; doi:10.26508/lsa.202301923)
Supplement: Supplementary file 1 [file LSA-2023-01923_TableS1.docx]

| MSMEG ID | Uniprot ID | Protein Description | Log2 fold change | Adjusted P-Value |
| --- | --- | --- | --- | --- |
| MSMEG_5489 | A0R3I9 | 50S ribosomal protein L32 | 1.83 | 0.0009 |
| MSMEG_0713 | A0QQD1 | Transcriptional regulator, MerR family protein (HspR) | 1.56 | 0.0046 |
| MSMEG_2686 | A0QVT3 | Uncharacterized protein | 1.54 | 0.0009 |
| MSMEG_1293 | A0QRZ5 | Xanthine/uracil permeases family protein | 1.50 | 0.0012 |
| MSMEG_2344 | A0QUV1 | Dehydrogenase | 1.34 | 0.0398 |
| MSMEG_6722 | A0R6Z2 | Uncharacterized protein | 1.33 | 0.0008 |
| MSMEG_2262 | A0QUM6 | Hydrogenase-2, small subunit | 1.31 | 0.0153 |
| MSMEG_2262 | A0QUM5 | Uncharacterized protein | 1.31 | 0.0104 |
| MSMEG_2112 | A0QU82 | Secreted protein | 1.30 | 0.0156 |
| MSMEG_6368 | A0R5Z4 | DNA-binding protein | 1.28 | 0.0011 |
| MSMEG_6059 | A0R543 | Uncharacterized protein | 1.23 | 0.0008 |
| MSMEG_6210 | A0R5J1 | Uncharacterized protein | 1.19 | 0.0279 |
| MSMEG_2619 | A0QVL8 | Efflux protein | 1.16 | 0.0158 |
| MSMEG_0202, MSMEG_0397, MSMEG_0802, MSMEG_1003, MSMEG_1059, MSMEG_1258, MSMEG_1404, MSMEG_2003, MSMEG_2283, MSMEG_2339, MSMEG_2828, MSMEG_3166, MSMEG_3697, MSMEG_4403, MSMEG_4790, MSMEG_4926, MSMEG_4945, MSMEG_5094, MSMEG_5378, MSMEG_6148, MSMEG_6155, MSMEG_6161, MSMEG_6462, MSMEG_6697 | A0QNX9 | IS1096, tnpA protein | 1.15 | 0.0415 |
| MSMEG_1782 | A0QTB6 | Oxidoreductase, short chain dehydrogenase/reductase family protein | 1.13 | 0.0203 |
| MSMEG_1794 | A0QTC7 | Dehydrogenase | 1.13 | 0.0253 |
| MSMEG_2582 | A0QVI1 | Uncharacterized protein | 1.05 | 0.0043 |
| MSMEG_5021 | A0R284 | Alcohol dehydrogenase, zinc-containing | 1.04 | 0.0024 |
| MSMEG_0987 | A0QR51 | Uncharacterized protein | 1.03 | 0.0295 |
| MSMEG_1758 | A0QT92 | Uncharacterized protein | 1.02 | 0.0188 |
| MSMEG_0918 | A0QQY3 | Transcriptional regulator, XRE family protein | 1.01 | 0.0012 |
| MSMEG_3710 | A0QYM2 | Cytochrome b561 family protein | 1.00 | 0.0236 |

**Table S1**
