# Supplementary material for: ATP-independent substrate recruitment to proteasomal degradation in mycobacteria
Source: Life Sci Alliance. 2023 Aug 10;6(10):e202301923. doi: 10.26508/lsa.202301923 (PMC10415612; doi:10.26508/lsa.202301923)
Supplement: Supplementary file 2 [file LSA-2023-01923_TableS2.docx]

| MSMEG ID | Uniprot ID | Protein Description | Log2 fold change | | Adjusted P-Value |
| --- | --- | --- | --- | --- | --- |
| MSMEG_0713 | A0QQD1 | Transcriptional regulator, MerR family protein (HspR) | 2.36 | 0.0018 | |
| MSMEG_4739 | A0R1G0 | Uncharacterized protein | 1.46 | 0.0234 | |
| MSMEG_1893 | A0QTM1 | UbiE/COQ5 methyltransferase | 1.42 | 0.0202 | |
| MSMEG_5221 | A0R2S9 | GCN5-related N-acetyltransferase | 1.37 | 0.0224 | |
| MSMEG_3395 | A0QXR3 | Short chain dehydrogenase | 1.34 | 0.0494 | |
| MSMEG_2589 | A0QVI8 | Uncharacterized protein | 1.29 | 0.0202 | |
| MSMEG_5279 | A0R2Y7 | Uncharacterized protein | 1.21 | 0.0224 | |
| MSMEG_1656 | A0QSZ5 | Exodeoxyribonuclease III | 1.14 | 0.0236 | |
| MSMEG_0613 | A0QQ36 | Uncharacterized protein | 1.11 | 0.0141 | |
| MSMEG_5562 | A0R3Q8 | Ribonuclease H | 1.08 | 0.0234 | |
| MSMEG_6591 | A0R6L6 | Aminotransferase, class V family protein | 1.04 | 0.0221 | |
| MSMEG_1854 | A0QTI2 | Valosin containing protein-1 | 1.04 | 0.0145 | |
| MSMEG_5216 | A0R2S4 | Glyoxalase family protein | 1.02 | 0.0350 | |

**Table S2**
