# Supplementary material for: ATP-independent substrate recruitment to proteasomal degradation in mycobacteria
Source: Life Sci Alliance. 2023 Aug 10;6(10):e202301923. doi: 10.26508/lsa.202301923 (PMC10415612; doi:10.26508/lsa.202301923)
Supplement: Supplementary file 3 [file LSA-2023-01923_TableS3.docx]

| **Peptide number** | **Residues of HspR** | **Amino acid sequence** |
| --- | --- | --- |
| 1 | 16-30 | VAAELAGMHAQTLRT |
| 2 | 31-45 | YDRLGLVSPRRTSGG |
| 3 | 46-60 | GRRYSLHDVELLRQV |
| 4 | 61-76 | QHLSQDEGVNLAGIK |
| 5a | 77-84 | RIIELTSQ |
| 5b | 85-91 | VEALQSR |
| 6 | 92-106 | LQEMAEELAVLRANQ |
| 7 | 107-121 | RREVAVVPKSTALVV |

**Table S3**
